# Nutritional Status and Obstacles to Healthy Eating Among Refugees in Geneva

**DOI:** 10.1007/s10903-020-01085-4

**Published:** 2020-09-17

**Authors:** Delphine Amstutz, Daniela Gonçalves, Patricia Hudelson, Silvia Stringhini, Sophie Durieux-Paillard, Sylvie Rolet

**Affiliations:** 1 Division of Primary Care Medicine, Department of Primary Care Medicine, Geneva University Hospitals, Geneva, Switzerland; 2 Unit of Population Epidemiology, Division of Primary Care Medicine, Geneva University Hospitals, Geneva, Switzerland; 3 Clinical Nutrition, Geneva University Hospitals, Geneva, Switzerland; 4 Care Directorate, Geneva University Hospitals, Geneva, Switzerland

**Keywords:** Migration, Refugees, Nutritional status, Healthy eating, Needs assessment

## Abstract

Refugees face various nutritional challenges during and after migration. This cross-sectional, mixed-methods study seeks to investigate the prevalence of undernutrition and obesity among refugees in Geneva, and to identify barriers to healthy eating. Anthropometric measurements of 354 adult refugees were collected between 2017 and 2019 by trained nurses and dietitians. Seven focus group discussions totaling 51 participants, refugees and social workers, investigated conceptions and needs regarding diet. The mean Body Mass Index is 24.6 ± 4.8 kg/m^2^. Women are disproportionately affected by obesity compared to men (p < 0.001). Weight gain post-migration is correlated positively with length of stay in Geneva (p < 0.001). Major obstacles to healthy eating are economic and linguistic. For participants, cooking workshops and free physical activities are highly needed interventions. Post-migration lifestyle interventions should be implemented to prevent weight gain in this population. Such interventions must be multi-level, to overcome structural, social and behavioral barriers to healthy eating.

## Background

Refugees face several nutritional challenges during their journey. They often arrive in the host country with protein-caloric deficiencies and/or micronutrient depletion [1–3]. This can be combined with an impaired initial nutritional status in their home country, as many regions now face a double burden of malnutrition [4]. In addition to migration itself, a range of structural and social determinants affect the diet and health of refugees, such as their socioeconomic position, housing conditions, cultural and language barriers or political factors [5].

Dietary acculturation, the process leading immigrants to adopt eating habits of the host country, is a complex process [6]. Systematic reviews examining acculturation among diverse ethnic groups in Europe have demonstrated that healthy aspects of the native diet (such as a high consumption of fruit, vegetables, whole grains and nuts) decrease while the consumption of foods rich in sugar, fat and salt increase [6, 7]. Dietary change over time among newly arrived immigrants depends on economic and time constraints, local language literacy and social support, among other factors [8].

While immigrants in Western countries have a lower BMI on arrival than native-born people, their risk to be overweight or obese slowly increases with their duration of stay, to match that of native people [9–12]. A young age at arrival seems to increase the risk of obesity [13], risk that also depends on the country of origin [11, 14].

In Switzerland, immigrants who have lived in the host country for at least 20 years are more likely to be overweight than Swiss nationals [15] and a study showed increased self-reported obesity and cardiovascular risk factors among immigrants [16]. African refugees themselves perceive a decreased consumption of fruit and vegetables and an increase in soft drinks post-migration [17]. However, to date, few studies have measured nutritional status among refugees and documented specific barriers to healthy eating.

In this paper, “refugees” include all people in the asylum process. The migratory wave of 2015–2016 increased the number of refugees by 33% in Switzerland [18]. In 2017, 6600 refugees were in Geneva, mainly young men from Eritrea, Afghanistan, Syria, Iraq, and Sri Lanka, living in community housing centers or individual dwellings [19]. In 2016, health workers from the Primary Care Division of the Geneva University Hospitals observed more micronutrient deficiencies and undernutrition in this population in their daily practice, while social workers noticed a rapid shift towards fast-food and unhealthy snacks. Consequently, in 2017, we launched a nutrition program in line with local, national and global action plans [20–22].

### Theoretical Frameworks

This study was guided by the WHO conceptual framework for action on the social determinants of health [23] and Liamputtong’s nutritional determinants of health [24]. The WHO conceptual framework illustrates how individual behaviors are influenced by the social position and structural determinants such as the broad political and socioeconomic context, including culture. The second framework explains how dietary individual choices are influenced by socioeconomic and cultural factors, themselves influenced by food availability, accessibility, affordability and marketing. Utilizing these concepts, we explored different levels of barriers to healthy eating among refugees.

### Aims

This baseline study assessed the nutritional situation of refugees in Geneva, to inform the design of a nutrition program. The study objectives were twofold: first, to investigate the prevalence of undernutrition, overweight and obesity among adult refugees; second, to identify barriers to healthy eating and needs regarding nutritional interventions.

## Methods

This is a cross-sectional, mixed methods study. Anthropometric and sociodemographic data were collected on a sample of refugees, and we conducted focus group discussions (FGDs) to explore barriers to healthy eating. Because all data were collected anonymously, our study does not fall within the scope of the Swiss law of the Human Research Act [25]. Although formal review of the study was waived by the Geneva Research Ethics Commission, we nonetheless obtained oral consent from all participants.

### Quantitative Data Collection

All data were collected on a voluntary basis. The sole exclusion criterion was being pregnant.

#### Participants and Settings for Data Collection

Between June 2017 and March 2019, trained nurses and dietitians measured anthropometric data (weight, height, and waist circumference) of 354 adults in several settings: hospital consultations, health centers and refugees’ housing sites. The sites of data collection were chosen on a practical basis, without randomization. We brought calibrated devices and stayed one day per site; all refugees present that day could participate. Several dates and times were proposed, to limit selection bias.

#### Measurements

Weight was measured using digital scales (with a level of precision of 100 g), with light clothes and no shoes, and height with a measuring rod, standing. Body Mass Index (BMI) was calculated as weight (kilograms) divided by height (meters)^2^ and classified following the WHO guidelines [26]. Waist circumference was measured between the top of the iliac crest and the bottom of the last palpable rib, after a normal exhalation [27]. Two measurements of each measure were taken and allowed to calculate the mean.

History of weight was self-reported. It included weight in the home country, evolution during migration and since arrival in Switzerland. Sociodemographic data included age, gender, country of origin, date of arrival and type of housing in Geneva.

Following data collection, individualized feedback was provided to participants, including brief nutritional advice and referral to healthcare professionals when appropriate.

### Qualitative Data Collection

Seven FGDs were conducted by two dietitians in different housing sites, between June and September 2017. We first purposively selected a variety of housing types (community housing centers, individual dwellings, and nuclear bunkers), to ensure a range of sociodemographic characteristics, since housing attribution is based on age, legal and civil status. We then recruited a convenience sample of residents at each site, with the help of social workers and flyers in various languages. Eligible participants were refugees who spoke French, English, Tigrigna or Tamil, and social workers. The latter have been included on the one hand to facilitate discussion, as the refugees identify and trust them, and on the other hand to provide a broad perspective about the living conditions of their residents.

A semi-structured topic guide was developed in French and covered: conceptions of a “healthy” diet, obstacles and facilitators to healthy eating, and perceived needs regarding nutritional interventions. Questions were based on theories about the social and nutritional determinants of health [23, 24]. Illustrations of potential barriers to healthy eating (e.g. money or language barrier) and examples of nutritional interventions (e.g. cooking workshops or gardening) were used to facilitate the discussion. Additional ideas evoked by participants were written on post-its. After brainstorming, participants were asked to individually prioritize three obstacles and three nutritional interventions needed by giving one point to each. Scores were then calculated based on all the FGDs to rank the main barriers and interventions needed.

Overall, 43 refugees from diverse origins, age and gender participated, together with eight social workers from different housing sites. Two FGDs were conducted in French, one in English and four with professional interpreters, in Tigrigna and Tamil.

One dietitian moderated the discussion while the other took detailed notes of what was said in French and noted nonverbal communication. Immediately after each FGD, the detailed description was completed by the observer, and reviewed by the moderator.

### Data Analysis

Descriptive statistics were performed using the R Statistical Software for quantitative data. Multiple linear regression analysis was performed; the final model correlated age, length of stay in Geneva and gender to explain BMI, weight gain and waist circumference since arrival in Geneva.

A descriptive and mainly deductive approach was used to analyze the FGDs [28]. Codes and themes were derived from the research questions: conception of a healthy diet, obstacles, facilitators, and needs regarding nutritional interventions. We also created an additional, inductive code to reflect spontaneous comments by participants about self-evaluation of their diets. Qualitative results were analyzed by the research team and two social scientists working with migrants.

## Results

### Sociodemographic Data

Of the 354 participants, 68.3% were men, mainly from Eritrea, Afghanistan, Sri Lanka and Syria (Table 1). Median length of stay in Geneva was 18 months, with important variations. A majority of participants lived in community housing centers.

**Table 1:** Sociodemographic characteristics of participants

Variables	Totals
Age, years (n = 348), mean ± SD	31.3 ± 9.6
Gender (n = 353), N (%)	
Men	241 (68.3%)
Women	112 (31.7%)
Country of origin (n = 354), N (%)	
Eritrea	97 (27.4%)
Afghanistan	42 (11.9%)
Sri Lanka	37 (10.5%)
Syria	37 (10.5%)
Iran	18 (5.1%)
Iraq	16 (4.5%)
Somalia	16 (4.5%)
Turkey	14 (3.9%)
Ethiopia	9 (2.5%)
Eastern Europe	9 (2.5%)
Others (mainly countries in central and north Africa, middle East and islands)	59 (16.7%)
Type of accommodation (n = 340), N (%)	
Community housing centres	273 (80.3%)
Underground nuclear bunkers	38 (11.2%)
Individual dwellings	29 (8.5%)
Length of stay in Geneva post-migration, months (n = 349), median (quartiles: P25–P75)	18 (1–24)
Newly arrived refugees (living ≤ 1 month in Geneva), N (%)	85 (24.4%)

### BMI

Mean BMI (n = 352) was trended towards the upper range for normal BMI: 24.6 ± 4.8 kg/m^2^, with 31.2% of participants being overweight (33.1% of women, 30.4% of men) and 10.8% obese (22.3% of women, 5.4% of men). Multiple linear regression analysis showed that BMI was correlated positively with age (p < 0.001) and significantly with gender, women having a higher mean BMI than men (26.4 kg/m^2^ and 23.7 kg/m^2^ respectively, p < 0.001) (Tables 2, 3). Table 3 presents gender stratified results.

**Table 2:** Association between sociodemographic factors and metabolic risk factors (multivariate analysis using R)

	BMI	Weight gain since arrival in Geneva	Waist circumference among women	Waist circumference among men
Age	0.154***	0.012	0.644***	0.525***
Length of stay in Geneva	0.010	0.103***	0.025	0.008
Gender (men)	− 2.218***	− 0.258	NA	NA

*p < 0.05, **p < 0.01, ***p < 0.001

**Table 3:** BMI, waist circumference and cardiovascular risk disaggregated by gender

	Men (n = 240)	Women (n = 112)	All (n = 352)
BMI (kg/m^2^)			
Mean ± SD	23.8 ± 4.0	26.4 ± 5.8	24.6 ± 4.8
Underweight (BMI ≤ 18.5), N (%)	12 (5.0%)	8 (7.1%)	20 (5.7%)
Normal weight (BMI 18.5–24.9), N (%)	142 (59.2%)	42 (37.5%)	184 (52.3%)
Overweight (BMI 25.0–29.9), N (%)	73 (30.4%)	37 (33.1%)	110 (31.2%)
Obese (BMI ≥ 30), N (%)	13 (5.4%)	25 (22.3%)	38 (10.8%)

	Men (n = 239)	Women (n = 103)
Waist circumference (cm)		
Mean ± SD	87.4 ± 10.4	91.1 ± 14.5
Median (quartiles: P25-P75)	87 (79–94)	91 (80–101)
Cardiovascular risk^a^		
Low cardiovascular risk, N (%)	186 (77.8%)	28 (27.2%)
Increased cardiovascular risk, N (%)	34 (14.2%)	17 (16.5%)
Substantially increased cardiovasc. risk, N (%)	19 (8.0%)	58 (56.3%)

^a^Categories based on WHO classification of waist circumference, for men: > 94 cm (increased risk) and > 102 cm (highly increased risk), for women: > 80 cm (increased risk) and > 88 cm (highly increased risk) [27]

Regarding height, the mean exactly matched the median: 1.72 m for men and 1.59 m for women, which suggests a similar history of socioeconomic status, nutritional status and growth during childhood within our sample [29].

### Waist Circumference

Table 3 shows that mean waist circumference was normal for men (87.4 ± 10.4 cm) and elevated for women (91.1 ± 14.5 cm). Waist circumference is correlated positively with age, for both genders (Table 2). It is recognized as a strong predictor of cardiovascular risk, risk of diabetes, hypertension and general mortality [27, 30–32]. In total, 8.0% of men and 56.3% of women had a substantially increased cardiovascular risk [27], having a waist circumference above 102 cm and 88 cm, respectively. Results might slightly underestimate abdominal obesity, as we took the general international cut-offs, while some populations (particularly African people), might have lower cut-off points [26, 30].

### Evolution of Weight During and After Migration

54 over 85 newly arrived refugees knew their weight in the country of origin. Two of them gained weight during migration, 28 maintained a stable weight, and 24 lost weight (on average, they lost 8.3 kg).

Among people having lived in Geneva for two months or longer at the time of measurement (n = 251), 201 knew their weight at their arrival. Of them, 50.7% had gained a minimum of 2 kg since their arrival (on average, 5.9 kg). The sole variable correlated significantly with weight gain was length of stay in Geneva (positive correlation, p < 0.001) according to the multiple linear regression analysis (Table 2).

### Conceptions of a Healthy Diet

Participants of the FGDs tended to define healthy diets by the type of foods consumed. Diets rich in fruit, vegetables, fish, poultry, starches (e.g. rice) and nuts, with little fats and few added sugars were considered healthy. A varied diet, having regular meals daily, good hygiene principles, and eating non-transformed foods without pesticides were also valued.

We need natural things, without chemicals. Organic foods. Fresh vegetables. You must avoid preservatives and pay attention to [food] conservation. (50-year-old, male refugee)

There were conflicting opinions about the healthiness of red meat and milk; some participants highlighted the need to eat them regularly while others thought they should be avoided.

### Self-assessment of Dietary Habits

In nearly all FGDs, participants self-assessed their dietary habits after explaining their ideas about healthy eating. Personal habits were either perceived positively or negatively, rarely neutral. Participants were proud of themselves if they ate fruit, vegetables, starches, poultry or fish regularly. Many participants considered they had a monotonous diet and used too much oil, some of them skipped meals and ate fast foods frequently, which they perceived as bad habits.

Sometimes it's good, because I have fruit, vegetables and starches in quantity, and sometimes it's not that good, because I use a lot of peanut paste and oil. (40-year-old, female refugee)

### Obstacles to Healthy Eating

**Fig. 1:**
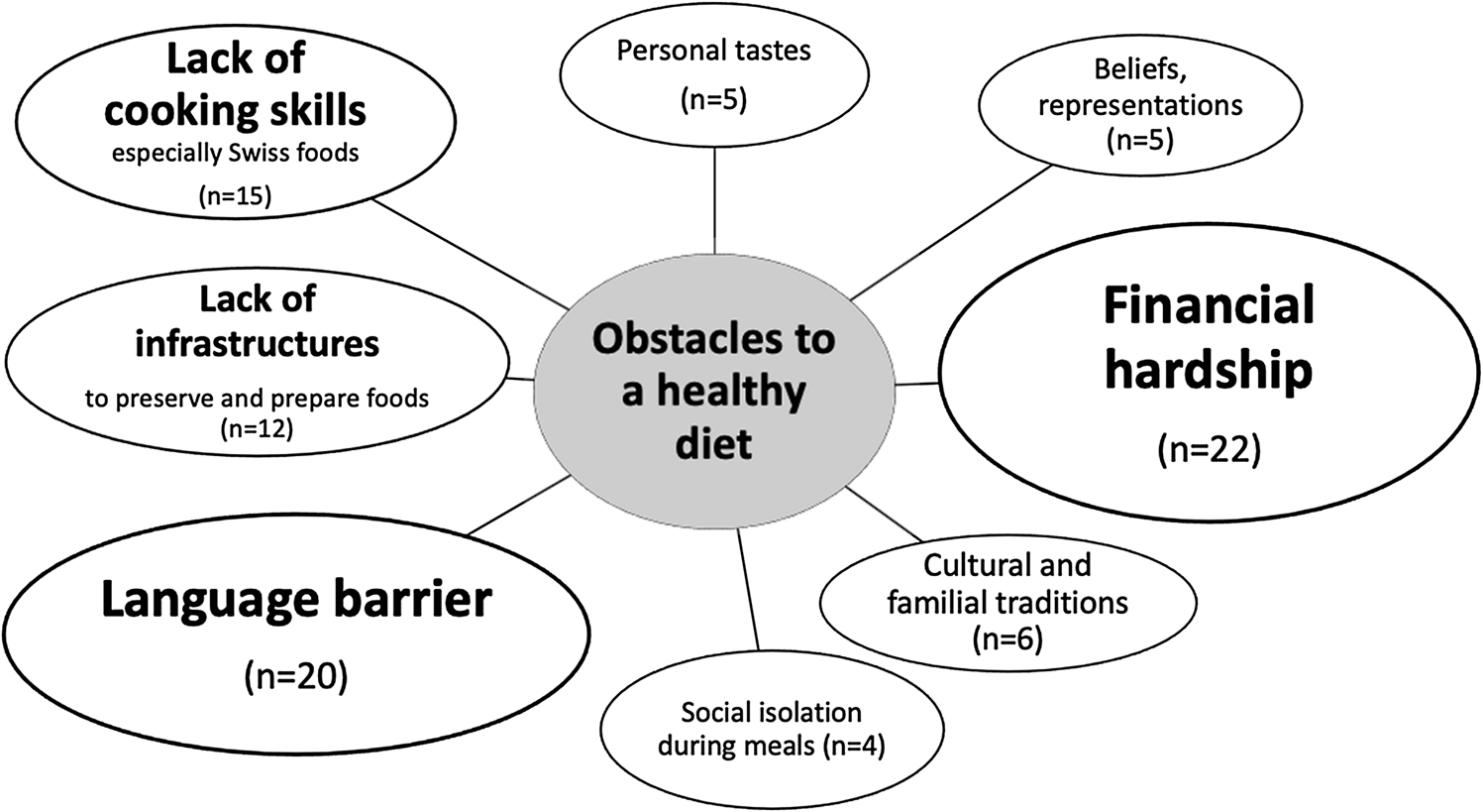
Obstacles to a healthy diet

Figure 1 illustrates the main obstacles mentioned by refugees and social workers. The more frequently an obstacle was cited, the larger the bubble (n represents the number of citations).

Most participants agreed that the main problem was the cost of “healthy” foods (especially fruit, vegetables, fish and meat), although a few participants said their budget was sufficient to eat a balanced diet. Unprocessed foods were valued, in parallel with the need to acquire new culinary skills, especially among young men:

Because of the lack of money, we do not control our diet. It's hard to have enough protein and vitamins. We eat for instance during three- or four-days rice or pasta, just that. And I do not know how to cook fish or chicken.[…] In fact, with our budget, we favor quantity over quality. (20-year-old, male refugee)

The most important thing is to eat vegetables, but I don’t know how to cook them. (25-year-old, male refugee)

The language barrier limited participants’ ability to understand food labels or to find familiar, traditional foods. The lack of adequate cooking facilities in some housing accommodations was also mentioned.

It's not easy for those who live in dormitories. They often eat one meal per day. There are also budget differences depending on legal status. I also note that depending on the country of origin, people do not know Swiss vegetables. Regarding African vegetables, they are very expensive here and loaded with pesticides. (30-year-old, female social worker)

In the beginning *(NB at arrival in Geneva)*, it was hard in the food shops, to ask, to lose time in order to find adequate foods. (62-year-old, female refugee)

Other obstacles were personal food preferences (e.g. for high-caloric foods), beliefs and traditions, or lack of pleasure in cooking alone. Some participants who lived in nuclear bunkers and received ready-made Swiss menus mentioned the lack of pleasure and cultural connection as a major obstacle.

### Facilitators

Several factors helped to achieve a healthy diet. First, having health problems that required dietary change (such as diabetes, hypertension, obesity) acted as a motivator, and frequent contact with healthcare professionals provided support for change. Second, having enough financial and housing autonomy to manage one’s budget and cook oneself was helpful. Having a family was also a motivating factor, especially for women. Finally, having learned to cook in the home country or finding community support in the host country was considered helpful. For instance, some community members showed newly arrived refugees where to shop and how to cook locally available foods.

### Needs for Nutritional Interventions

Refugees expressed a desire to receive professional advice about dietary recommendations and how to attain them with a small budget, to learn practical skills such as shopping and cooking, and to benefit from free sports and exercise activities. Social workers highlighted other priorities; to increase refugees’ budgets so that they could afford healthier foods and to encourage communal meals that would reinforce social bonds.

Nutritional intervention strategies which received higher scores were cooking workshops, physical activity sessions, general nutritional education, visits to (super-) markets, and advice on managing a small budget. These preferences inform the development of a future nutrition program.

## Discussion

This study assessed nutritional status and explored obstacles to healthy eating among refugees in Geneva. Anthropometric measurements of 354 adult refugees indicated a relatively low prevalence of underweight (5.7%) compared to what we expected after some long migratory routes with impaired food security, but this prevalence of undernutrition is still higher than among young Swiss adults (4.7%) [33]. Conversely, the prevalence of overweight and obesity was high in our sample, 42% overall. Those results changed the orientation of the nutrition program following this study, from fighting undernutrition to tackling the double burden of malnutrition, especially obesity.

The mean BMI, in the upper norm (24.6 kg/m^2^), was similar in this study and in the French-speaking Swiss population (24.8 kg/m^2^) [33]. However, our findings show important differences between genders: overweight and obesity affected over 55% of women (vs 36% of men) while 73% of females had abdominal obesity (vs 22% of men). In recent Swiss studies, it was the opposite: overweight and obesity affected 51% of men and 33% of women [34]. Cardiovascular risk factors were either more prevalent in men [35] or around 30% for both genders [33].

Our results are nevertheless consistent with BMI of refugees and immigrants worldwide. Massad et al. found that 76% of refugee women in Palestine are overweight, a rate much higher than in Palestinian women [36]. Similarly, almost 70% of Bhutanese refugee women in the United States [37] and 51% of Sudanese immigrants in Australia are overweight [38].

A significant correlation was found between length of stay since arrival in Geneva and weight gain, similarly to other studies [11, 39, 40]. Several factors might explain this, from the low socioeconomic status, to sedentary lifestyles and food insecurity. Some barriers to healthy eating could have been existing before migration, while being exacerbated by the rapid nutrition transition, once low-cost and high-caloric foods are suddenly easily accessible [36, 37, 41].

Financial hardship was the main barrier to healthy eating, in refugees and social workers’ perceptions. Linguistic difficulties, lack of cooking skills (especially Swiss foods) and sub-optimal infrastructures also explained a diet often considered by refugees themselves as monotonous. These results are consistent with other studies among refugees, which have shown that low income and limited language proficiency are linked to food insecurity [42, 43]. In comparison, in the Swiss population, barriers to healthy eating include daily routines, time constraints and personal preferences while the price is also the major obstacle [44].

Conception of a healthy diet was homogeneous and largely corresponds to the Swiss nutritional guidelines [45], despite some conflicting beliefs regarding meat and milk. Participants considered a diet rich in fruit and vegetables, based on fresh and untransformed foods as healthy whereas they perceived too much oil and junk foods negatively. This knowledge of nutritional recommendations by refugees is in line with other findings [46]. Several factors facilitated a healthy diet, such as concern about health, having a family, community support, and autonomy.

The need regarding nutritional interventions were different for refugees and social workers. Refugees mainly expressed the needs to receive dietary counseling given a small budget, to learn cooking skills, and access free physical activities. Social workers mentioned two priorities: increase refugees’ budget for foods and foster social cohesion through shared meals. This socioeconomic perspective is actually complementary to the practical demands expressed by refugees themselves. Burge and Dharod have found similar results: nutrition interventions among refugees should cover topics such as the role of a healthy diet, food shopping and food budgeting [47]. Culturally relevant nutrition programs, in particular programs co-created with the refugees themselves, are highly needed [48].

Previous studies exploring diet in the Swiss population have shown an overconsumption of meat and processed foods, an underconsumption of fruit and vegetables [34] and a lack of awareness about the food pyramid, especially among men and people with a low education level [33]. Furthermore, diet quality is known to contribute to socioeconomic inequalities in obesity [49]. Nutrition policies and programs should therefore address in parallel the needs of the refugees and of the native Swiss population. Our study highlights the need for multi-level nutrition programs, to tackle the structural, socioeconomic and behavioral determinants of obesity. Such programs must be flexible, rapidly proposed after resettlement and continuously adapted to the nutritional status and needs of the target population. Nutritious foods, consistent with the diversity of cultures represented in Switzerland, should be affordable and encouraged for all.

We have to acknowledge some limitations. First, the cross-sectional design and the number of missing data regarding history of weight make the big picture of the evolution of weight unclear. Another bias is the convenience sample regarding anthropometric measurements, that could hamper the generalizability of the findings: in our sample, countries of origin differ slightly from the general refugee population in Geneva (Eritrean and Sri Lankan overrepresented, Afghan and Syrian underrepresented), and people living in individual dwellings were underrepresented [18, 19]. They could face different challenges linked to nutritional status.

Regarding the FGDs, the researchers’ background (dietitians) induces a social desirability bias, which we tried to reduce with a neutral, empathic posture. The selection of four languages to conduct the FGDs does not allow representativeness of all ethnic groups of refugees. Another issue inherent to cross-cultural research is the information bias due to the translation of participants’ native language into French, then into English for publication purposes. However, the descriptive rather than interpretive nature of the study, and the collaboration with professional transcultural interpreters minimized this risk. Finally, the number and variety of respondents strengthens the validity of our findings.

### New Contribution to the Literature

This is the first study measuring nutritional status and exploring obstacles to healthy eating among refugees in Switzerland at this scale. Contrary to our first hypothesis, anthropometric measurements indicated a relatively low prevalence of underweight (5.7%) and a high prevalence of overweight and obesity (42%, like Swiss residents). Obesity affected women disproportionately: 55% of them were overweight or obese and 73% had an increased waist circumference.

Barriers to healthy eating include a range of structural, social and behavioral factors, inherent to the multiple determinants of health affected by migration and the rapid nutrition transition. Multi-level lifestyle interventions must be implemented to tackle obesity among refugees rapidly once they arrive in the host country. Finally, longitudinal studies should explore the mechanisms preventing or fostering weight gain after migration and nutrition programs should be evaluated to highlight their active ingredients.

## Data Availability

The dataset generated and analysed in this study is published via Université de Genève, Yareta repository (10.26037/yareta:wjapipqdr5eefatqcxatfts2ca). It will be preserved for 10 years.

## References

[1] Benson J (2013). Low vitamin B12 levels among newly-arrived refugees from Bhutan, Iran and Afghanistan: a multicentre Australian study. PLoS ONE.

[2] Meziani C (2012). The double burden of obesity and malnutrition in a protracted emergency setting: a cross-sectional study of western sahara refugees. PLoS Med.

[3] Ackerman LK (1997). Health problems of refugees. J Am Board Fam Med.

[4] WHO. The double burden of malnutrition: policy brief. WHO/NMH/NHD/17.3. 2017. https://apps.who.int/iris/bitstream/10665/255413/1/WHO-NMH-NHD-17.3-eng.pdf?ua=1. Accessed 16 Feb 2018.

[5] Marmot M, Allen J, Bell R, Bloomer E, Goldblatt P, Consortium for the European Review of Social Determinants of Health and the Health Divide (2012). WHO European review of social determinants of health and the health divide. Lancet.

[6] Satia-Abouta J, Patterson RE, Neuhouser ML, Elder J (2002). Dietary acculturation: applications to nutrition research and dietetics. J Am Diet Assoc.

[7] Gilbert PA, Khokhar S (2008). Changing dietary habits of ethnic groups in Europe and implications for health. Nutr Rev.

[8] Patil CL, Hadley C, Nahayo PD (2009). Unpacking dietary acculturation among New Americans: results from formative research with African refugees. J Immigr Minor Heal.

[9] Goel MS, McCarthy EP, Phillips RS, Wee CC (2004). Obesity among US immigrant subgroups by duration of residence. J Am Med Assoc.

[10] Oza-Frank R, Cunningham SA (2010). The weight of US residence among immigrants: a systematic review. Obes Rev.

[11] McDonald JT, Kennedy S (2005). Is migration to Canada associated with unhealthy weight gain? Overweight and obesity among Canada’s immigrants. Soc Sci Med.

[12] Rhodes CM, Chang Y, Percac-Lima S (2016). Development of obesity and related diseases in african refugees after resettlement to united states. J Immigr Minor Heal.

[13] Roshania R, Narayan KMMV, Oza-Frank R (2008). Age at arrival and risk of obesity among US immigrants. Obesity.

[14] Lindström M, Sundquist K (2005). The impact of country of birth and time in Sweden on overweight and obesity: a population-based study. Scand J Public Health.

[15] Office fédéral de la statistique (OFS). Enquête Suisse sur la santé. 2019. https://www.bfs.admin.ch/bfs/fr/home/statistiques/sante/etat-sante/migrants.html. Accessed 29 Jan 2020.

[16] Grossmann FF, Leventhal ME, Auer-Böer B, Wanner P, Bischoff A (2011). ‘Self-reported cardiovascular risk factors in immigrants and swiss nationals. Public Health Nurs.

[17] Kruseman M (2005). Post-migration dietary changes among African refugees in Geneva: a rapid assessment study to inform nutritional interventions. Soz Praventivmed.

[18] Secrétariat d’Etat aux Migrations. Statistique en matière d’asile 2016. 2017. https://www.sem.admin.ch/dam/data/sem/publiservice/statistik/asylstatistik/2016/stat-jahr-2016-kommentar-f.pdf. Accessed 5 Apr 2017.

[19] Hospice général (2017). Statistiques mensuelles—mars 2017.

[20] Etat de Genève. Concept cantonal de promotion de la santé et de prévention 2030. 2016. www.ge.ch/concept-psp. Accessed 8 Jan 2019.

[21] OFSP. Stratégie nationale de prévention des maladies non transmissibles. 2016. https://promotionsante.ch/assets/public/documents/fr/2-pgv/Strategie_MNT_2017-2024.pdf. Accessed 15 Jan 2019.

[22] UN. Sustainable development goals. 2018. https://sustainabledevelopment.un.org/sdgs. Accessed 10 May 2018.

[23] WHO. A conceptual framework for action on the social determinants of health. 2010. https://apps.who.int/iris/bitstream/10665/44489/1/9789241500852_eng.pdf. Accessed 18 Feb 2018.

[24] Liamputtong P (2016). Public health: local and global perspectives.

[25] Swiss Confederation. Federal act on research involving human beings (human research act). 2014. https://www.admin.ch/opc/en/classified-compilation/20061313/index.html. Accessed 10 May 2019.

[26] OMS. Obésité et surpoids: principaux faits. 2018. https://www.who.int/fr/news-room/fact-sheets/detail/obesity-and-overweight. Accessed 12 Mar 2019.

[27] WHO (2011). Waist circumference and waist-hip ratio: report of a WHO Expert Consultation.

[28] Sandelowski M (2000). Whatever happened to qualitative description?. Res Nurs Health.

[29] Garn SM (1975). Nutrition, growth, development, and maturation: findings from the ten-state nutrition survey of 1968–1970. Pediatrics.

[30] Han TS, Van Leer EM, Seidell JC, Lean MEJ (1995). Waist circumference action levels in the identification of cardiovascular risk factors: prevalence study in a random sample. BMJ.

[31] Huxley R, Mendis S, Zheleznyakov E, Reddy S, Chan J (2010). Body mass index, waist circumference and waist:hip ratio as predictors of cardiovascular risk. Eur J Clin Nutr.

[32] Pouliot M (1994). Waist circumference and abdominal sagittal diameter: best simple anthropometric indexes of abdominal visceral adipose tissue accumulation and related cardiovascular risk in men and women. Am J Cardiol.

[33] Bochud M (2017). Anthropometric characteristics and indicators of eating and physical activity behaviors in the Swiss adult population: results from menuCH 2014–2015.

[34] Office fédéral de la statistique (OFS) (2020). Santé: Statistique de poche 2019.

[35] Firmann M (2008). The CoLaus study: a population-based study to investigate the epidemiology and genetic determinants of cardiovascular risk factors and metabolic syndrome. BMC Cardiovasc Disord.

[36] Massad SG (2018). Metabolic syndrome among refugee women from the west bank, Palestine: a cross-sectional study. Nutrients.

[37] Batta M, Assad L, Shakya S (2014). Socio-demographic and dietary factors associated with excess body weight and abdominal obesity among resettled Bhutanese refugee women in Northeast Ohio, United States. Int J Environ Res Public Health.

[38] Renzaho AMN, Bilal P, Marks GC (2014). Obesity, type 2 diabetes and high blood pressure amongst recently arrived Sudanese refugees in Queensland, Australia. J Immigr Minor Heal.

[39] Cairney J, Ostbye T (1999). Time since immigration and excess body weight. Can J Public Heal.

[40] Goel MS, Mccarthy EP, Phillips RS (2004). Obesity among US immigrant subgroups by duration of residence. J Am Med Assoc.

[41] Popkin BM (2011). Contemporary nutritional transition: Determinants of diet and its impact on body composition. Proc Nutr Soc.

[42] Vahabi M, Damba C, Rocha C, Montoya EC (2011). Food insecurity among Latin American recent immigrants in Toronto. J Immigr Minor Heal.

[43] Gallegos D, Ellies P, Wright J (2008). Still there’s no food! Food insecurity in a refugee population in Perth, Western Australia. Nutr Diet.

[44] de Mestral C, Stringhini S, Marques-Vidal P (2016). Barriers to healthy eating in Switzerland: a nationwide study. Clin Nutr.

[45] Société Suisse de Nutrition. La pyramide alimentaire suisse. 2019. https://www.sge-ssn.ch/fr/toi-et-moi/boire-et-manger/equilibre-alimentaire/pyramide-alimentaire-suisse/. Accessed 16 Jun 2019.

[46] Tiedje K (2014). A focus group study of healthy eating knowledge, practices, and barriers among adult and adolescent immigrants and refugees in the United States. Int J Behav Nutr Phys Act.

[47] Burge C, Dharod JM (2018). What are the nutrition education needs of refugees: assessment of food choices, shopping and spending practices of South-Asian refugees in the USA. J Int Migr Integr.

[48] Wieland ML (2012). Physical activity and nutrition among immigrant and refugee women: a community-based participatory research approach. Women’s Heal Issues.

[49] de Mestral C, Chatelan A, Marques-Vidal P, Stringhini S, Bochud M (2019). The contribution of diet quality to socioeconomic inequalities in obesity: a population-based study of Swiss adults. Nutrients.

